# A unified model of Hymenopteran preadaptations that trigger the evolutionary transition to eusociality

**DOI:** 10.1038/ncomms15920

**Published:** 2017-06-23

**Authors:** Andrés E. Quiñones, Ido Pen

**Affiliations:** 1 Theoretical Research in Evolutionary Life Sciences, Groningen Institute for Evolutionary Life Sciences, University of Groningen, P.O. Box 11103, 9700 CC Groningen, The Netherlands

## Abstract

Explaining the origin of eusociality, with strict division of labour between workers and reproductives, remains one of evolutionary biology’s greatest challenges. Specific combinations of genetic, behavioural and demographic traits in Hymenoptera are thought to explain their relatively high frequency of eusociality, but quantitative models integrating such preadaptations are lacking. Here we use mathematical models to show that the joint evolution of helping behaviour and maternal sex ratio adjustment can synergistically trigger both a behavioural change from solitary to eusocial breeding, and a demographic change from a life cycle with two reproductive broods to a life cycle in which an unmated cohort of female workers precedes a final generation of dispersing reproductives. Specific suits of preadaptations are particularly favourable to the evolution of eusociality: lifetime monogamy, bivoltinism with male generation overlap, hibernation of mated females and haplodiploidy with maternal sex ratio adjustment. The joint effects of these preadaptations may explain the abundance of eusociality in the Hymenoptera and its virtual absence in other haplodiploid lineages.

## Abstract (web summary)

The Hymenoptera are thought to have preadaptations responsible for the repeated evolution of eusociality in ants, bees, and wasps. Here, Quiñones and Pen model the evolution of primitively social insects and identify factors that synergistically promote the transition to eusociality.

Eusociality characterized by physically differentiated castes is the most advanced form of social life found in the animal kingdom. Its hallmark is reproductive division of labour, where some individuals (workers) refrain from their own reproduction but instead increase the reproductive output of their parents. The first evolutionary hypothesis that attempted to explain the distribution of eusociality across animal taxa, the ‘haplodiploidy hypothesis’, was proposed by Hamilton using his then new inclusive fitness theory^1^,^2^. Haplodiploidy is the sex determining mechanism whereby fertilized eggs become females, and unfertilized eggs males. As a consequence, full sisters are genetically more closely related to one another (*r*=3/4) than to their own daughters (*r*=1/2), thus favouring workers to channel more effort into raising the former rather than the latter. When Hamilton proposed his hypothesis, almost all the taxa known to have evolved eusociality—with the notable exception of termites^3^—were in the haplodiploid Hymenoptera, the order of ants, bees and wasps. Thus, it seemed at least plausible that haplodiploidy was an important preadaptation: a trait that arose before, and independently of evolving helpers at the nest, but that appeared to increase the chances for facultative helping to ultimately produce a sterile worker caste. However, since then a number of additional diploid eusocial clades have been discovered, including ambrosia beetles^4^, sponge–dwelling shrimp^5^ and bathyergid mole rats^6^, suggesting that the apparent importance of haplodiploidy is less obvious^2^,^7^. The workers in these newly discovered diploid eusocial clades, despite showing reproductive altruism, have maintained their capacity to become reproductively active, at least under certain circumstances. In contrast, the advanced eusocial clades—all of them haplodiploid aculeate Hymenoptera except the termites—have obligate reproductive altruism; that is, the workers never mate and have lost their reproductive totipotency. Thus, haplodiploidy might still have an important facilitating role in the evolution of advanced stages of eusociality.

Trivers and Hare^8^ pointed out that Hamilton’s original model overlooked the fact that in haplodiploid species females are more closely related to their sons (*r*=1/2) than their brothers (*r*=1/4), and that this exactly cancels the genetic benefits of helping raise siblings instead of helping to raise offspring. Consequently, only when the production of the sexes is split among different nests (split sex ratios), will haplodiploidy favour worker behaviour, and it will do so only in those nests with female-biased sex ratios^8^,^9^,^10^. Thus, maternal ability to bias offspring sex ratios came to be seen as an additional preadaptation for the evolution of eusociality in Hymenoptera, in addition to any environmental conditions that would favour split sex ratios^10^,^11^. Seger showed that temporally split sex ratios are promoted by the bivoltine life cycle found in many species of solitary insects closely related species with helpers at the nest^11^. Bivoltinism, the production of two non-overlapping broods in one reproductive season, opens the possibility to split the production of the sexes between the two broods. Seger^11^ hypothesized that a female-biased second brood later in the season would promote the evolution of helping behaviour in the first brood, thus adding bivoltinism as yet another preadaptation for the early evolution of eusociality. A female-biased summer brood implies that if a spring-hatched female stays to help her mother, she would help raise siblings that are on average more closely related to her than her own offspring would be, if she would assist in raising an even sex ratio. However, Seger’s argument pertains only to the initial invasion of the helping trait, and does not account for possible changes in sex ratios driven by the presence of helping behaviour, once it has evolved^12^. Sex allocation theory predicts evolutionary feedbacks between helping behaviour and sex ratios when only one of the sexes helps, as is the case in Hymenoptera^13^,^14^,^15^, thus making it necessary to jointly account for the co-evolution of helping behaviour and facultative sex ratio adjustment.

Besides haplodiploidy, maternal manipulation of offspring sex ratios and bivoltinism, several additional factors have been proposed to bias the odds in favour of eusociality, such as specific life cycle structures, ecological conditions^16^,^17^ and last but not least a monogamous mating system^18^,^19^,^20^. However, it is unknown how these factors jointly affect the evolution of reproductive altruism, and in particular whether they act synergistically in promoting it. Here we develop a unified model, grounded in the life history of primitively social insects described by Seger^11^. The model integrates many of the proposed preadaptations, and allows the co-evolution of helping behaviour and sex ratios. We show that, indeed, specific combinations of traits, life history characteristics and ecological conditions strongly increase the likelihood that reproductive altruism evolves. Furthermore, we show that sex ratio evolution causes the production of a first brood of unmated workers before a brood of reproductives, leading to a univoltine life cycle reminiscent of annual colonies of bumblebees and vespine (yellowjacket) wasps that produce workers in the spring and early summer and a final brood of reproductive at the end of the season.

## Results

### Partial bivoltinism

**Figure 1:**
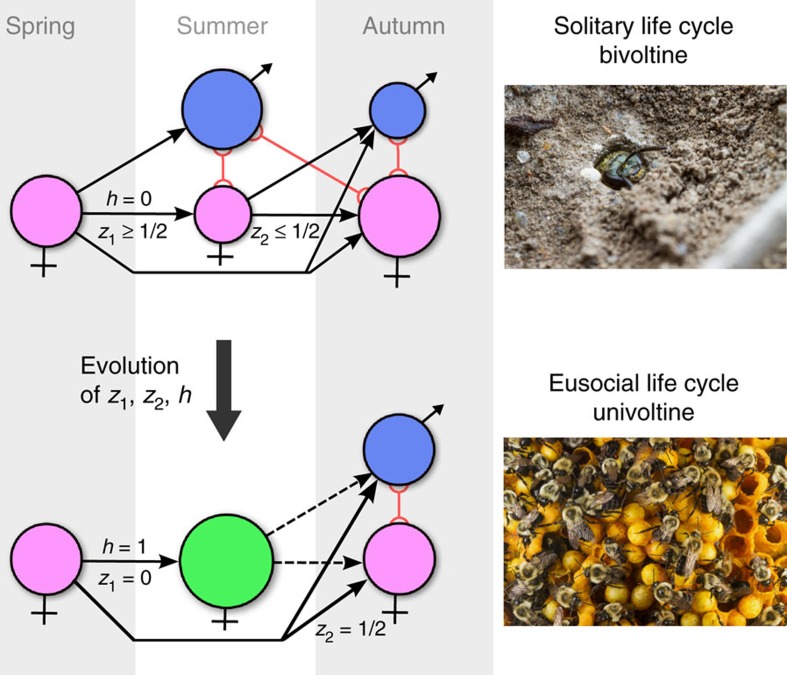
Life cycles at the beginning and end of the evolutionary transition. Partially bivoltine solitary life cycle used in the model as evolutionary starting point (top) and univoltine eusocial life cycle obtained as evolutionary endpoint (bottom). Disks depict different classes of individuals: pink and blue disks are female and male reproductives, respectively, while the green disk represents female workers. Black arrows represent contribution from one class to another via reproduction (filled) and helping behaviour (dashed). Red lines connect male classes with potential mates in female classes. Evolvable parameters in the model are the spring sex ratio *z*_1_, the summer sex ratio *z*_2_ and the helping tendency *h* of female offspring hatched in the spring. Top: each spring starts with females that mated during the previous autumn, survived hibernation and founded a new nest. Each overwintering female can produce up to two broods per year: one in the spring and one in the summer, giving rise to, respectively, broods of summer and autumn adults. Females from spring broods reproduce once during the summer. Males from spring broods mate with females from spring broods and can also survive to mate in autumn with females from summer broods. Before helping evolves (*h*=0), selection favours male-biased spring sex ratios (*z*_1_>1/2) and female-biased summer sex ratios (*z*_2_<1/2)^11^. Bottom: an evolutionarily derived effectively univoltine life cycle as it evolves when the partially bivoltine life cycle in the top diagram is increasingly characterized by retaining helper daughters at the nest. At the end point if this development, only unmated females are produced during spring (*z*_1_=0) and these females help their mother (*h*=1) raise the summer brood, which has an unbiased sex ratio (*z*_2_=1/2). Photographs Alex Wild, used by permission.

We constructed models for populations with two partially overlapping generations per year, that is, partially bivoltine populations that are common in Hymenoptera^11^ (details in the Methods—Demography). The first generation of the year, or spring generation, gives rise to a summer generation consisting of offspring and survivors of the spring generation. The summer generation then gives rise to an autumn generation, some of which members overwinter to form a new spring generation, thus completing the life cycle (Fig. 1, top). In the models, females have three potentially evolvable traits: the probability 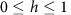 of females hatching in the spring to forgo reproduction and stay at their natal nest to help their mother (for example, individuals with *h*=0 never stay to help; of individuals with *h*=0.5, half stay to help while the other half leave), and the offspring sex ratios: (proportion of sons) 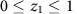 and 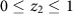 produced in spring and summer broods, respectively. Each helper adds an additional *B* offspring for each offspring produced by her mother. *B>*1 implies that helpers are more efficient at raising siblings than at raising their own offspring, while *B<*1 means that they are more efficient at raising their own offspring than they are at raising siblings.

### Unisexual or bisexual overwintering

We followed Seger^11^ in considering two types of partially bivoltine life cycle: the ‘female hibernation’ (FH) type where only mated females from the autumn generation overwinter, and the ‘larval diapause’ (LD) type where both sexes overwinter as diapause larvae^10^,^11^ (Supplementary Figs 1 and 2). For both types of cycle, overwintering females reproduce first in the spring, and may reproduce (if they survive) a second time during summer, while females from the spring brood can only reproduce in the summer. In the FH model, males hatched in the spring can mate with females hatched in the spring, and if they survive (with probability *S*_m_) they can mate with females hatched the in the summer as well. In contrast, males hatched in the summer can only mate with females hatched in the summer. As a result, in the FH scenario, males hatched in the spring have an inherently higher expected reproductive success than males hatched in the summer. This causes natural selection to favour male-biased sex ratios in the spring and female-biased sex ratios in the summer^11^. In the LD model the situation is reversed in that males from the summer brood overwinter and get the chance to mate both in the following spring, with overwintering females, and, if they survive (with probability *S*_m_), once again in the summer with females from spring broods; in contrast, males from the spring brood mate only in the summer with females from the spring brood. Thus, the LD life cycle favours male-biased sex ratios in the summer and female-biased sex ratios in the spring^11^.

### Eusociality threshold

We modelled the life cycles described above using the matrix population model approach. We first derived a transition matrix to track the dynamics of rare mutant phenotypes invading a resident population monomorphic for the three traits. Using the reproductive value approach^21^,^22^, we derived the selection gradient for each of the traits. Our main interest was in studying the conditions under which altruistic helping (*h*>0) evolves, and how this is affected by the presence or absence of specific preadaptations. To this end, we analysed different versions of our models, by varying: (1) the life cycle structure that depends on whether only females overwinter (FH), or both males and females overwinter (LD, see previous section for details); (2) the type of genetics (diploidy or haplodiploidy); (3) the mating system, with several alternative options (obligate monandry=lifetime monogamy with life-time storage of sperm from a single ejaculate, polyandry with females storing sperm of a variable number of males (*m*_*e*_; see Methods—Polyandry) for life, serial monogamy with females needing to re-mate and store new sperm for producing a second brood; and lastly, (4) the presence or absence of maternal sex ratio control, that is, the ability to produce different offspring sex ratios for the first and the second broods. We analysed the effects of the preadaptations by deriving the minimal level of helper benefits (*B*_min_) necessary for selection to favour the evolution of helping; we refer to this quantity as the eusociality threshold. This measure is equivalent to the ‘efficiency ratio’ of Charnov^23^ and Grafen^9^, and the ‘potential for altruism’ of Gardner^24^,^25^, the relative efficiency of a helper in raising sibs as opposed to own offspring at which she is indifferent between those two options. Finally, we computed evolutionary dynamics of these traits using an adaptive dynamics approach^26^,^27^ based on our inclusive fitness expressions^28^, and we complement them with matching individual-based population genetic simulation models (see Methods—Individual-based simulations for details).

### Co-evolutionary dynamics

**Figure 2:**
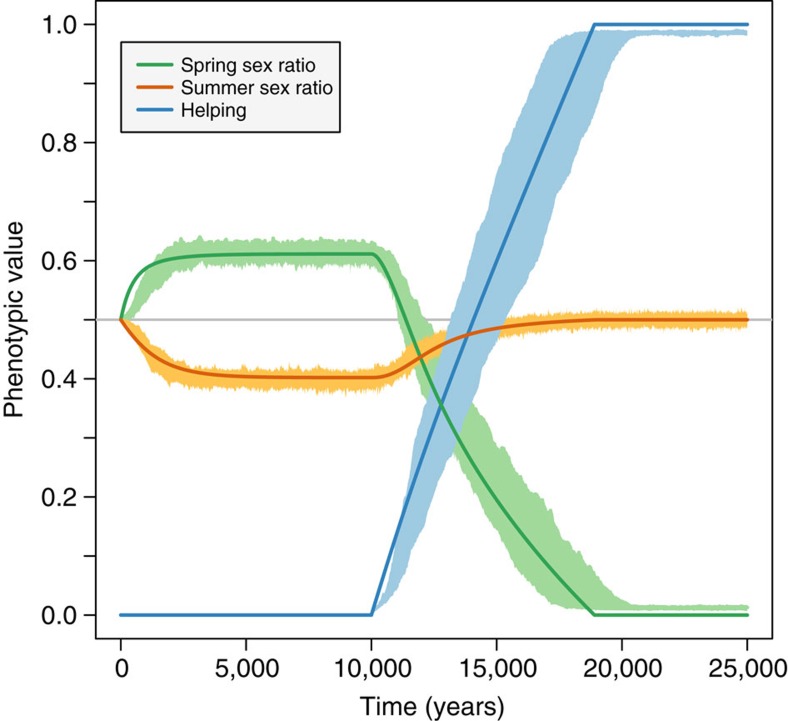
Coevolutionary dynamics driving the evolutionary transition. Helping behaviour (blue), spring (green) and summer (yellow) sex ratios coevolved in populations with a female hibernation life cycle, haplodiploid genetics and lifetime monogamy. Smooth darker curves are deterministic predictions from the mathematical inclusive fitness model; more lightly coloured ribbons represent corresponding outcome ranges of 10 stochastic individual-based simulations. During the first 10,000 generations, helping is not allowed to evolve, while sex ratios evolve towards male-biased spring broods and female-biased summer broods. From generation 10,000 onwards, helping is allowed to evolve: initial evolution of helping behaviour feeds back on sex ratio selection, reversing the direction of selection on sex ratios; as helping evolves towards maximal levels (*h*→1), implying that all first brood offspring have become workers, spring broods evolve towards 100% female sex ratios (*z*_1_→0), while summer broods evolve towards an even sex ratio (*z*_2_→1/2). At evolutionary equilibrium, a transition has occurred from a partially bivoltine life cycle without helping behaviour towards a social univoltine life cycle with the production of a first brood of unmated workers followed by a second brood of reproductives with an even sex ratio. Parameter values *S*_f_=0.9, *S*_m_=0.6, *b*=1.5, *F*_1_=*F*_3_=5.0.

An analysis of the co-evolutionary dynamics of helping and sex ratios shows that the FH life cycle, with overlapping generations of males, initially favours the evolution of male-biased broods in the spring and female-biased broods in the summer (Fig. 2), as predicted by Seger^11^. Thus, sex ratio manipulation under the FH life cycle strongly promotes a transition from solitary breeding to reproductive altruism, while the LD life cycle tends to inhibit it (Methods—Selection on helping behaviour). Moreover, once helping behaviour is present and daughters increase the fecundity of their mother, natural selection favours mothers that allocate more resources into the production of more helpers. This is achieved by shifting the sex ratio of the spring brood to produce more females. Eventually, the spring brood becomes 100% female helpers which, due the complete lack of males in their cohort, all remain unmated and thus represent the start of an obligate worker caste. Moreover, the same lack of spring males causes the summer sex ratio to evolve back to fifty–fifty (Fig. 2). This amounts to a major life history transition from a partially bivoltine (Fig. 1, top) to a univoltine life cycle cycle, with a specialized breeder and life-time unmated workers (Fig. 2, bottom), triggered by an evolutionary feedback between social behaviour and sex ratios^12^. This evolutionary feedback is robust to assumptions in the mutational structure of the three evolving traits (Supplementary Fig. 3).

### Preadaptations and synergies in the evolution of helpers

**Figure 3:**
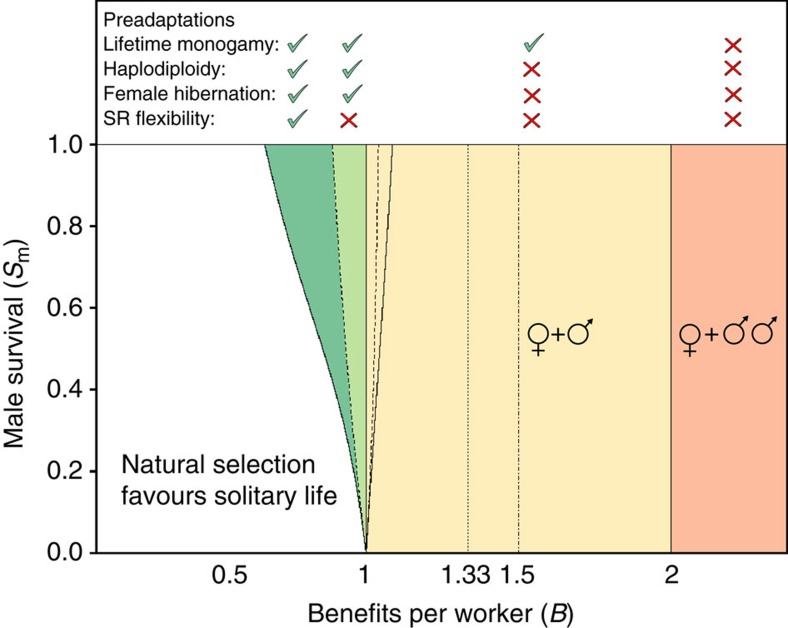
Effect of preadaptations and synergies in the evolution of reproductive altruism. Helping efficiency *B* required for the evolution of eusociality as a function of male survival probability from first (spring) to second (summer) brood (*S*_m_), partially bivoltine life cycle type (female hibernation versus larval diapause), maternal ability to adjust her brood sex ratios (present versus absent), type of mating system (monogamy versus various forms of polyandry), and genetical system (haplodiploidy versus diploidy). A helping efficiency of *B*=1 indicates that a worker is equally efficient at raising her own offspring as she is at helping her mother raise offspring. Under serial monogamy (single random mate per brood) or extreme polyandry (many mates per brood) eusociality always requires workers to be at least twice as efficient raising siblings relative to own offspring (orange area, *B*>2). Under strict monogamy and diploidy, eusociality always requires workers to be more efficient at raising siblings than their own offspring (yellow area, *B*>1). Intermediate cases of female polyandry are shown by dotted (effective number of mates per female equals two requires *B*>1.33) and dot-dashed (effective number of mates per female equals three requires *B*>1.5) lines (see Methods—Polyandry hampers the evolution of helping). Under the combination of strict monogamy, haplodiploidy and larval diapause, the efficiency required for the evolution of helpers increases with male survival (dashed line inside yellow area), and more strongly if sex ratios can coevolve (full line inside yellow area). Under strict lifetime monogamy, haplodiploidy and female hibernation, the required benefits decrease with male survival (dashed line in green area), more strongly so if sex ratios can coevolve (left border of dark green area). Top panel: green check marks (red crosses) indicate presence (absence) of preadaptations on the left of the top panel and referring to the differently coloured areas in the diagram. Parameter values *F*_1_=*F*_3_=2.0.

Our models, unlike Hamilton’s original haplodiploidy hypothesis^1^, account for the joint effects of genetics (ploidy) and life history/ecological traits (overwintering, mating system, sex ratios, sex-specific survival). However, the relative importance of the different traits differs in magnitude and consistency. Lifetime monogamy^18^, for instance, due to its positive effect on within-brood genetic relatedness, unambiguously reduces the eusociality threshold to one half of the threshold under serial monogamy and up to one half of the threshold for varying degrees of polyandry (Fig. 3). In contrast, haplodiploidy^15^ and sex ratio manipulation can both favour and harm the evolution of helping behaviour^25^, depending on the specific life cycle (FH or LD, Fig. 3). However, the simultaneous presence of lifetime monogamy, haplodiploidy, and sex ratio manipulation in a FH life cycle can reduce the threshold to only 2/3 of their productivity as solitary breeders (Fig. 3, Methods—Selection on helping behaviour). Specifically, for haplodiploids with an FH life cycle, where females on average have an effective number of mates^29^ *m*_e_ and use the sperm of the same males for both broods, the condition for altruistic helping behaviour to be favoured by selection is


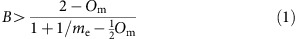


Here 0≤*O*_m_≤1 is a measure of generation overlap between males born in the first brood and males born in the second brood (see Methods—Class-specific individual reproductive values). If no males from the first brood mate with females from the second brood (*O*_m_=0), then (1) reduces to *B*>1 at best (monogamy: *m*_e_=1) and *B*>2 at worst (extreme polyandry: *m*_e_→∞). Conversely, if males from the first brood monopolize females from the second brood (*O*_m_→1), as a result of strongly split sex ratios between males in the first brood and females in the second brood, then (1) reduces to *B*>2/3 at best (*m*_e_=1) and again *B*>2 at worst (*m*_e_→∞). The right-hand side of (1) strictly increases with the effective number of mates *m*_*e*_; therefore, any increase in polyandry (higher *m*_e_), all else being equal, makes it harder for altruistic helping behaviour to evolve (Supplementary Fig. 4).

For diploids, the equivalent condition is


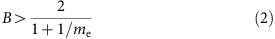


Note that the right-hand side of (2) is always larger than the right-hand side of (1) and never smaller than one and that every deviation from monogamy (*m*_e_>1) will make helping behaviour less likely to evolve. For the LD life cycle, the corresponding conditions (1) and (2) are always less favourable for the evolution of reproductive altruism (see Methods—Selection on helping behaviour).

Combining the results for haplodiploids and diploids, in order for the threshold value of *B* to dip below unity, the necessary conditions are haplodiploidy, female hibernation, partial survival of spring males until the summer cohort hatches (*O*_m_>0) and limited promiscuity 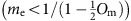; together these conditions are sufficient. Thus, in taxa possessing combinations of specific preadaptations (lifetime monogamy, haplodiploidy, sex ratio manipulation and unisexual overwintering), the likelihood of the evolution of altruistic helping is significantly increased. Note that this does not imply the evolution of a caste of unmated workers. Coevolution of sex ratios and helping behaviour leads eliminates the production of males in the spring brood (Fig. 2, z1→0), and with this, the end of male generation overlap (*O*_m_→0; see Methods—Demography). Thus, the final step of the evolutionary transition to a specialized unmated worker caste requires the benefits of helpers to be larger than 1.

## Discussion

The two types of life history considered here represent distinctive characteristics found in different taxonomic groups of Hymenoptera. The female hibernation life cycle is a common feature of Halictine bees^30^, bumblebees^31^,^32^, Vespine and *Polistes* wasps^33^, where despite large diversity in life history and social behaviour, life cycles usually start with a solitary mated female or (in *Polistes*) occasionally groups of mated females. Overwintering characterizes the life cycle of many temperate species due to seasonality, but tropical species often also have periods of inactivity driven by the cycles of rainy and dry weather^34^. Halictine bees are the group of animals in which helper reproductive altruism has evolved more times than in any other group^35^. In primitively eusocial Halictines, despite workers keeping the potential to reproduce, the life cycle resembles the one described by our model: colonies first produce a female-biased helper brood followed by a reproductive brood at the end of the season. In contrast, the initial stage in our model, although present, is rare among solitary Halictines^30^. Most solitary Halictine species produce only a single brood per reproductive season. Some species, such as *Halictus sexcinctus* and *H. rubicundus*, can both nest solitarily and with helpers depending on their geographical location; solitary populations are univoltine, but if they live in locations where the length of the season allows more than one brood, they recruit daughter helpers as described by our model^36^,^37^. The rareness of the bivoltine life cycle and the corresponding facultative sex ratios, both in solitary and social Halictines, has been used as an argument against the broader applicability of Seger’s model to understand the evolution of helper and worker caste^25^,^38^. Our model resolves that issue by showing that strict bivoltinism and a female-biased summer brood are no longer expected once helpers become unmated workers, due to the feedback between sex ratio evolution and the helping behaviour. Turning the argument upside down, we expect the bivoltine life cycle with female hibernation to be rare in groups with helping behaviour and female-biased summer broods. In agreement with our model predictions, comparative analysis has shown that a female-biased sex ratio in the first brood correlates positively with the degree of sociality across insect lineages with helpers at the nest^39^,^40^.

The larval diapause condition is found in sphecid wasps, for which—although less well studied—there is only a single report of helpers at the nest^41^. However, there is some evidence of the sex ratio biases predicted by the Seger model^11^,^42^. Larval diapause is also common among Megachilidae bees^43^, the almost exclusively solitary sister lineage of the corbiculate bees (honey bees, stingless bees, bumblebees, orchid bees)^44^.

Lifetime monogamy is a preadaptation that extends beyond the Hymenopteran scenario portrayed by our model. The favourable effects that lifetime monogamy has on the evolution of reproductive altruism and cooperative breeding have both considerable theoretical^20^,^25^,^45^,^46^ and empirical support. Strict parental monogamy has now been well documented as the ancestral state across lineages of the social Hymenoptera and termites with permanent worker castes^47^,^48^, but the mechanisms are very different. In the Hymenoptera lifetime monogamy is facilitated by the presence of lifetime sperm storage^18^,^48^, which could be considered a morphological preadaptation. In higher termites, lifetime monogamy is facilitated by the royal chamber inside the nests^18^,^48^. In our model, any departure from lifetime monogamy reduces the prospects for the emergence of reproductive altruism, and the presence of monogamy enhances the favourable effects of haplodiploidy and sex ratio manipulation on the likelihood of retaining helpers at the nest. Our analysis also shows that strict monogamy is not necessary for the threshold value of the benefits per helper *B* to be smaller than unity; for sufficiently low levels of polyandry, the combined presence of haplodiploidy, female hibernation, sex ratio manipulation and male generation overlap may cause the threshold *B*<1 facilitating the evolution of reproductive altruism (Supplementary Fig. 4). However, in the final stages of the evolutionary transition, due to the lack of males, strict monogamy is necessary for the threshold to be as low as *B=*1.

Some recent models have questioned the general positive effect of monogamy for the evolution of eusociality^49^,^50^. However, a more general analysis has shown that putative exceptions regarding the effect of monogamy are restricted to cases where altruism is determined uniquely by a single allele of strong effects^51^. Moreover, these models focus on the rate of initial increase of alleles for helping behaviour–where multiple mating by males carrying mutant alleles allows for faster spreading of mutant alleles across colonies–but not on equilibrium levels of altruism after multiple successive invasions of mutant alleles of smaller effects^49^,^50^. Our models, in contrast, assume that gradual substitution of many alleles of small effect is driving the evolution of social behaviour, consistent with empirical data on life-history traits being generally polygenic and heritable in a quantitative genetics sense^52^. Thus, we believe that the evidence, both theoretical and empirical, supports the role of monogamy as being crucial for the evolution of a worker caste.

Apart from the specific predictions about sex ratios in different broods, our model highlights the importance of the production of more than one brood per year. The production of two broods opens the possibility to have opposite sex ratio biases in each one of them, a form of temporal split sex ratios^10^,^11^. Bivoltinism has also been proposed as crucial for the origin of a developmental preadaptation that favours evolution of the worker phenotype^17^,^53^. The origin of helper and worker phenotypes requires a developmental system that expresses reproductive altruism at the right moment of the life cycle^54^,^55^. Bivoltinism requires tuning the expression of two metabolically different phenotypes: one that stores reserves and enters into hibernation or aestivation diapause (summer brood), and one that reproduces immediately (spring brood)^56^. The developmental system that tunes the expression of these two distinctive bivoltine phenotypes could then be co-opted to express the worker and queen phenotypes^17^,^53^. Thus, multiple sources of evidence point towards bivoltinism as a predecessor a facilitating factor for retaining helpers at the nest. Multivariate statistical analysis shows that indeed the number of broods, governed by breeding season length and developmental time, is positively correlated with the level of sociality among facultative and obligately eusocial species^57^. Moreover, environmental factors related to season length appear to be important determinants for the expression of helping behaviour in species that can either have helpers or be solitary, depending on geographical location^36^,^37^,^58^,^59^.

Hamilton’s idea that haplodiploidy favours the evolution of reproductive altruism has been challenged on both empirical and theoretical grounds^7^,^8^,^10^,^25^,^46^. The empirical grounds are that reproductive altruism occurs also in several diploid species^7^. However, up until now there has been no formal phylogenetically corrected statistical analysis to assess whether eusociality is more often found in haplodiploid lineages than diploid ones; but, a quick look still seems to suggest there is^60^. The theoretical grounds are that when both sexes are properly accounted for in the inclusive fitness calculations, haplodiploidy requires female-biased sex ratios to favour eusociality^8^. However, recent models suggest that the conditions (polyandry, see above) which favour such biased sex ratios are unlikely to have been present in the early evolution of eusociality^25^,^45^,^46^. Here we have shown that, depending on the type of bivoltine life cycle, haplodiploidy can both inhibit and promote the evolution of reproductive altruism. When the bivoltine life cycle starts with mated females emerging from hibernation, haplodiploidy strongly favours the evolution of helpers at the nest. Furthermore, haplodiploidy is a sex determination system that allows flexibility in maternal control of offspring sex ratios. Given that we identified maternal sex ratio manipulation to be another preadaptation, haplodiploidy has a two-fold effect in the evolution of reproductive altruism in the Hymenoptera. This does not mean that our model predicts in general that helpers at the nest evolved more often in species with haplodiploid genetics; but rather, that reproductive altruism will be found more often in species with haplodiploid genetics that present also the other preadaptations. Furthermore, a closer look at species with diploid genetics might lead to the discovery of preadaptations specific to that case^19^.

The evolutionary scenario encapsulated by our model starts with a population living in a seasonal environment that allows the production of two broods, associated with two mating episodes in one reproductive season. Our model predicts that evolution drives such populations towards a single synchronous mating episode in the life cycle, a condition predominant in all taxa with obligatorily unmated workers, even when living in tropical environments and with secondarily evolved obligate polyandry in that single episode^48^. Once a univoltine life cycle with a first brood of workers has evolved, it is possible to envision evolutionary, demographic and ecological changes that extend the specialization of the first (worker) brood, leading eventually to physically differentiated castes. For starters, under the predicted fully female first brood, females from the first brood cannot mate. Thus, reproductive competition inside the nests does not compromise the social endeavour, paving the way for further adaptation of females to their worker role. The evolution of fully committed workers, which have lost reproductive totipotency^18^, increases the benefits they provide to the colony. Such more elaborate commitments requires drastic behavioural and developmental changes, during the large number of generations required to decisively modify developmental pathways towards queen and worker phenotypes^18^,^48^. For seconds, environmental changes that increase the length of the reproductive season may allow the production of a larger worker brood. These changes could occur simply by range expansion toward lower latitudes or altitudes. A larger worker brood would then possibly enable colonies to gather enough resources to transition towards a perennial life cycle. Evidence for such a transition can be found in bumblebees, where temperate species invariably have the univoltine eusocial life cycle predicted by our model, while some tropical relatives have a perennial life cycle, while maintaining the female hibernation condition and a single-synchronous mating episode^32^. However, as long as unmatedness of the first brood females has not gone to fixation, reductions in the length of the breeding season may reinstate solitary life, which appears to have happened more often in Halictines than in any other lineages with helpers at the nest^35^. Yet other evolutionary scenarios might be driven by conflicts inside the colony; for example, queen-worker conflict over control of the sex ratio of the second brood, or conflict over worker production of male eggs^61^, might lead to alternative social organizations and life histories. Taking into account those potential scenarios will probably give a better prediction of the distribution of eusociality in the Hymenoptera.

The evolutionary transition to eusociality, the most advanced form of social life, encompasses radical and complex changes in many facets of a species’ biology. Rather than one unique causal factor, we showed how specific combinations of them can drive the transition. Monogamy, haplodiploidy with maternal sex ratio manipulation, bivoltinism with male generation overlap, and hibernation of mated females combine to provide the most favourable conditions for the evolution of reproductive altruism. The Hymenoptera seem to have serendipitously ended up with such a set of traits, and because of them, have achieved their supreme ecological position.

## Methods

### General modelling approach

We consider populations with two partially overlapping generations per year, that is, partially bivoltine populations with a spring generation and a summer generation. Each generation has a specific class structure, that is, a frequency distribution of females and males, reproductives and helpers. The vector 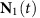 keeps track of the class distribution for the spring generation in year *t*, while 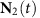 tracks the summer generation in year *t*. Transitions between generations are modelled with demographic matrices 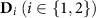 that track survivors and the offspring produced by females^62^:


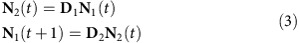


At demographic equilibrium (DE; when 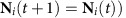, all transitions in a single year can be described by a block matrix of the form^63^


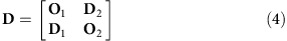


The **O**_i_ are matrices of appropriate dimensions filled with zeroes. The dominant eigenvalue of **D** must be *λ*=1. To ensure this we scale winter survival by a factor *α* (see ‘Demography’ below). The corresponding dominant right eigenvector **u** contains for both generations the stable class distributions in DE, which will be needed for the inclusive fitness calculations outlined below.

Class-specific individual reproductive values, the long-term genetic contributions of individuals to future generations^64^, are derived from a gene flow matrix. This matrix keeps track of female and male reproduction and survival, where each contributor to a newly born or survivor in the next time step gets credit according to the proportion of genes derived from the contributor. For example, in haplodiploids a female gets 100% credit for her male offspring, while females and males both get 50% credit for each of their female offspring. In diploids both parents get 50% credit for offspring of both sexes. The gene flow matrices have the same block structure as the demographic matrices:


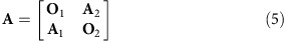


The dominant left eigenvector **v** of **A**, that is, the solution of 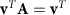, where *T* denotes transposition, contains the class specific individual reproductive values.

To model the evolution of a trait *x*, where *x* is a sex ratio or helping tendency, we analysed the inclusive fitness of a focal mutant individual with trait *x* in a resident population fixed for trait value x*. If the focal individual belongs to class *k*, and contributes 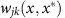 individuals to class *j*, with average relatedness 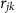 to the focal individual, then the focal individual’s inclusive fitness is given by


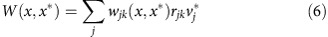


where 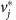 is the reproductive value of class-*j* individuals in the resident population^28^. The selection differentials with respect to *x* are then given by


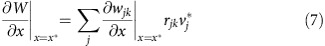


If a trait is expressed by individuals in more than one class *k*, then the selection differentials are obtained by summing over the appropriate classes, weighing each class according to its normalized class frequency 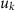.

Evolutionary equilibria are calculated by setting the selection differentials to zero and solving for x* Evolutionary dynamics are modelled using a standard adaptive dynamics approach^27^, where the rate of change of x* over evolutionary time is proportional to the selection differential:


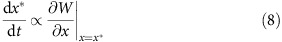


We used individual-based simulations to check the results of the analytical inclusive fitness analyses; details of the simulations are in section Individual based simulations.

### Demography

Here we construct the demographic models that keep track of the class frequencies in the partially bivoltine populations. A summary of our notation is in Table 1. We consider two kinds of life history: the Female Hibernation (FH; Supplementary Fig. 1) life history and the Larval Diapause (LD; Supplementary Fig. 2) life history. For the moment, we assume female lifetime monogamy, i.e. each female mates once in her life with a single male; later (section ‘Polyandry hampers the evolution of helping’) we drop this assumption.

### Demography for female hibernation

We keep track of seven classes of individuals (Supplementary Fig. 1). The spring generation consists of mated females, the ‘foundresses’, which have survived hibernation (class 1) and the sperm they carry (class 2). Each mated female produces *F*_1_ offspring, a fraction 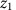 of which are sons (class 7) and 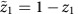 daughters (classes 3 and 4). A fraction *h* of daughters remain at the natal nest and become helpers (class 4), while 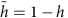 become independent breeders (class 3). Mated females themselves survive until summer to breed again with probability *S*_f_ (class 5), along with the sperm they still carry (class 6).

These transitions between spring and summer generations are encapsulated by the matrix


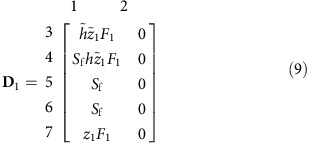


Numbers above the columns indicate contributing classes, numbers along rows indicates ‘receiving’ classes. Note that column 2 contains zeroes only since we only count offspring of females in order to prevent double counting.

In the summer generation, each class 3 female mates with a single class 7 male and produces *F*_3_ offspring, with sex ratio 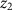. Each class 5 female, which may have helpers at her nest, produces *F*_5_ offspring with sex ratio 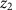. We sometimes refer to the offspring produced by the summer generation as the ‘autumn offspring’. These autumn offspring mate in the autumn, as do a fraction *S*_m_ of class 7 males of the summer generation. The mated females go into hibernation and have density-dependent survival such that the number of class 1 females in the next spring is the same as in the previous spring. These transitions are governed by the matrix


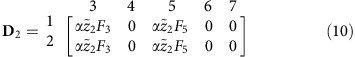


Both demographic matrices can be combined into a single matrix that keeps track of the class frequencies in both generations at DE:


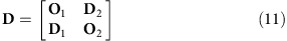


Here **O**_1_ and **O**_2_ are respectively 2 × 2 and 5 × 5 matrices filled with zeroes.The DE relative class frequencies are solutions of 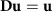 and they are


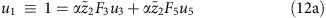



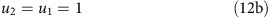



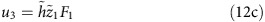



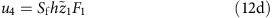



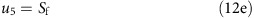



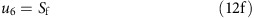



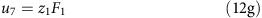


Note that we have set the relative frequency *u*_1_ of class 1 to unity and expressed the other class frequencies as multiples of the class 1 frequency. This normalization determines the density-dependent female winter survival, as follows:


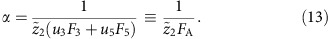


*F*_A_ can be regarded as the total number of autumn offspring per spring female, and 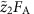 therefore the corresponding number of females among autumn offspring. The scaling ensures that on average every spring female is exactly replaced by another spring female one year later.

Given the DE class frequencies, the average number of mates per male during the autumn mating season, respectively during the summer mating season, are given by:


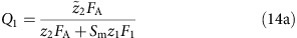



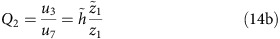


In the autumn, males produced by the summer generation 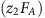 and the surviving males of the summer generation 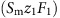 compete together for 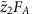 females, hence on average *Q*_1_ mates per male. In the summer, the class 7 males compete for the non-helping class 3 females, hence *Q*_2_ mates per male.

### Demography for larval diapause

For the LD life history, the spring generation consists of unmated females and males that survived winter diapause. This first generation mates randomly and produces the summer generation, consisting of surviving adults and new offspring (Supplementary Fig. 2). It is conceptually convenient to split up summer males into surviving spring males and the sons of spring females, hence we now have 8 classes in total. Following the same approach as for the FH life history, the DE class frequencies are tracked by the block matrix


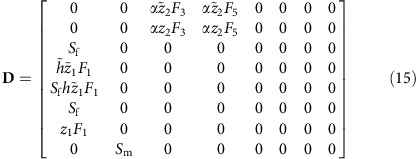


The stable class distribution follows again from 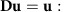



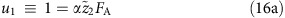



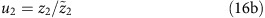



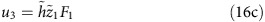



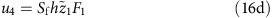



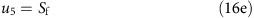



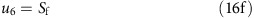



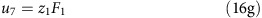



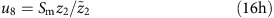


The average number of mates for spring and summer males are, respectively,


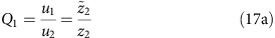



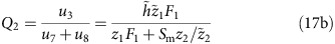


### Effect of helping

For both FH and LD life histories, the expected number of helpers for a class 5 summer female is given by


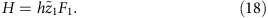


We assume that each helper increases the number of offspring produced by her mother by an amount 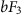, hence


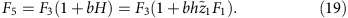


Note that a class 5 female’s output increases linearly with the number of daughters 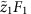 she produced during the spring.

At the moment a helper ‘decides’ to stay at her natal nest, her expected contribution *B* for each of her mother’s future offspring is conditional on her mother’s survival:


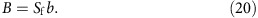


### Class-specific individual reproductive values

Individuals of different classes typically differ in their relative contributions to the future gene pool, and individual reproductive values (RVs) quantify these differences^64^. In matrix population models, the individual RVs are the elements of the dominant left eigenvector of a gene flow matrix, and they are used as weights in inclusive fitness calculations^28^,^65^. In this section, we first derive the class-specific RVs and then use them to derive the RVs of daughters and sons for the spring and summer generations.

### Reproductive values of haplodiploids with female hibernation

The gene-flow matrix is easily derived from Supplementary Fig. 1 by inspecting the outgoing edges from each node and determining what proportion of genes in a ‘receiving’ node can be attributed to the ‘donating’ node. Surviving individuals obviously contribute 100% of their genes to their surviving selves, while under haplodiploidy a male offspring derives 100% of his genes from his mother and for a female offspring both her mother and her father get credit for 50% of her gene content. For the haplodiploid FH life history, this gives rise to the following gene flow matrix:


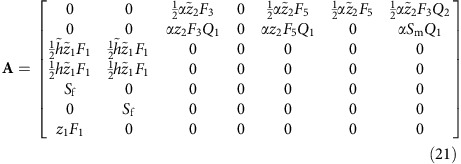


Note that the columns that correspond to contributions by males contain the 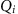 defined in equation (17a,b), which are the mean numbers of mates per male.The RVs are solutions of 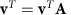:


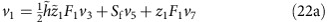



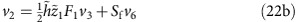



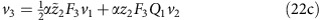



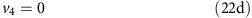



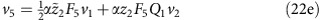



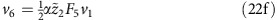



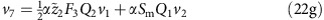


The RVs of daughters and sons born in the spring and the summer, respectively, can then be calculated as


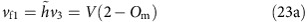



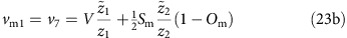



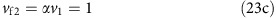



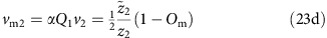


Here


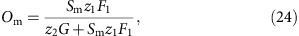



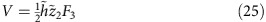


and


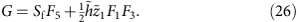


Note that the RVs (23a–d, 74, 75) are normalized such that the RV of a daughter born in summer, 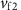, is set to unity and the other RVs are multiples of 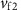. A crucial quantity is 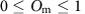, which can be regarded as measure of generation overlap between spring and summer males. If 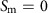 and/or 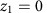, male generations do not overlap, and the relative within-generation RV of males to females reduce to the familiar 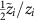 for haplodiploids^66^. On the other hand, if there is male generation overlap (*O*_m_>0), then in the spring the RV of males increases with respect to that of females, while in the summer the RV of males decreases with respect to that of females. This explains selection for male-biased spring sex ratios and female-biased summer sex ratios for the FH life cycle, at least as long as helping is rare (see section ‘Sex ratios selection’).

### Reproductive values of diploids with female hibernation

The gene-flow matrix differs from the haplodiploid case in that both parents now get credit for 50% of male offspring:


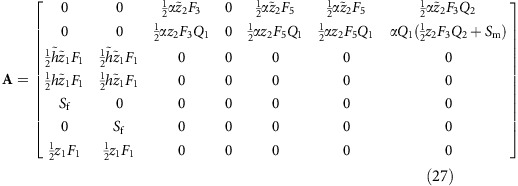


The class-specific RVs are then given by:


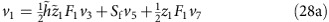



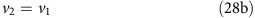



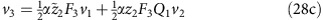



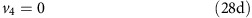



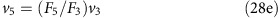



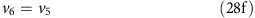



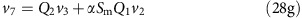


The spring and summer RVs of daughters and sons are now


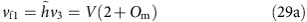



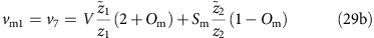



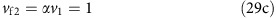



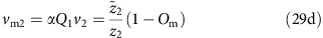


Here


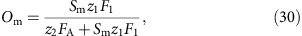


is again a measure of the degree of generation overlap in males. It is slightly smaller than corresponding quantity for haplodiploids; thus, in diploids the divergence in RVs between females and males will be slightly smaller as well. If there is no male generation overlap, that is 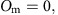 then the relative RVs of males to females reduces to the familiar 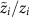 for diploids^66^.

### Reproductive values of haplodiploids with larval diapause

The gene-flow matrix can be derived from Supplementary Fig. 2, as follows:


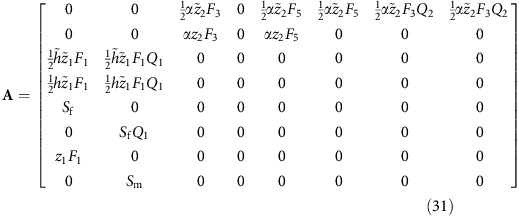


The corresponding RVs are given by


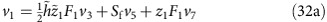



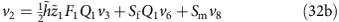



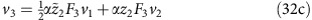



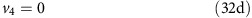



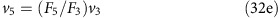



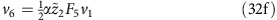



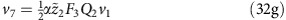



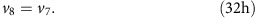


The RVs of daughters and sons in spring and summer are now given by


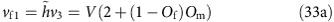



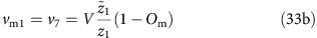



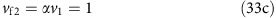



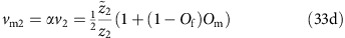


Here


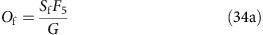



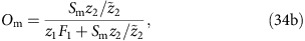


are measures of generation overlap in females and males, respectively. If 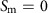, there is no overlap between male generations, and the relative RVs of males to females again reduces to the familiar 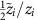 for haplodiploids. In contrast to the case for the FH life cycle, for the LD life cycle male generation overlap reduces the RV of spring males relative to that of spring females, while the RV of summer males is increased with respect to that of summer females. This explains why selection favours female-biased spring sex ratios and male-biased summer sex ratios (see ‘Sex ratios selection’).

### Reproductive values of diploids with larval diapause

The gene-flow matrix is now given by


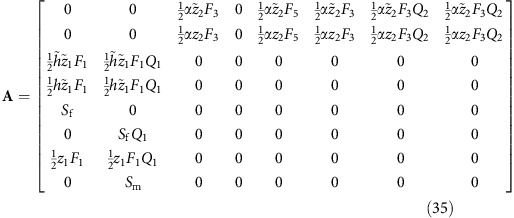


The RVs are then given by the equations


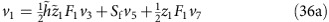



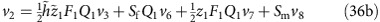



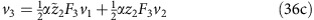



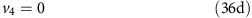



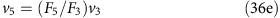



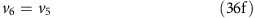



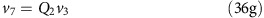



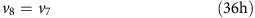


The RVs of daughters and sons in spring and summer are now given by


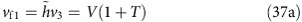



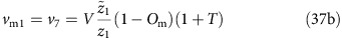



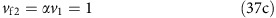



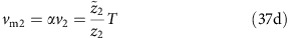


Here male generation overlap 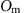 is given by equation (34b) and


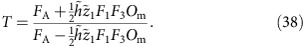


Note that 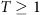 with equality if there is no male generation overlap. As before, if there is no male generation overlap, that is, 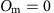, then the relative RVs of males to females reduces to the familiar 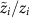 for diploids.

### Sex ratios selection

In this section, we derive inclusive fitness expressions and corresponding selection differentials for females controlling the spring sex ratio 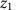 and summer sex ratio 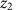.

The inclusive fitness expressions are a focal female’s expected lifetime number of daughters and sons, each weighed by a corresponding coefficient of relatedness and RV, given the focal female’s sex ratios 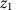 and 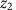 in a resident population with sex ratios 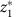
and 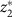:


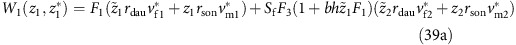



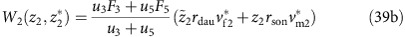


Variables equipped with an asterisk are evaluated at their resident values 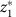 and 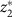. In outbred populations, the relatedness of a daughter to her mother is 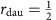, for both haplodiploids and diploids, while a son is related to his mother by 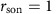 for haplodiploids and 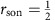 for diploids. The first term in the inclusive fitness (37a) of a spring female correspond to her *F*_1_ offspring produced in the spring, while the second term corresponds to her expected number of offspring 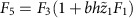 produced in the summer, provided she survives with probability 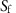. Note that her total inclusive fitness increases with the number of female helpers, given by equation (18), which in turn increases with the proportion of daughters 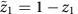 produced in the spring.The corresponding selection differentials are given by:


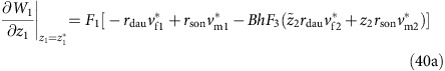



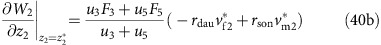


Note that in the absence of help (*h*=0), the sex ratios 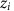 are in equilibrium if and only if daughters and sons yield the same ‘life-for-life’ relatedness to their mother: 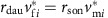. Otherwise, all else being equal, the presence of female helpers shifts selection in the spring towards more daughters.

### Sex ratio selection in haplodiploids with female hibernation

Plugging in the reproductive values (23a–d, 74, 75) and the relatedness coefficients, the selection differential (40a) for 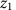 can be written as


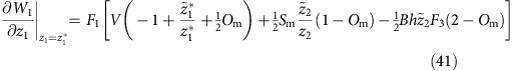


The parameter *O*_m_ is given by equation (24) and measures overlap of male generations. It is easy to see that in the absence of help (*h*=0), and with non-overlapping generations of males 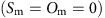, all terms between brackets but the first vanish and the equilibrium sex ratio is 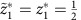. Without help but with overlapping male generations 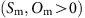, the equilibrium spring sex ratio is male-biased, that is, 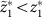. With help (*h*>0), the last term on the right of equation (41) may come to dominate and a female-biased sex ratio may be favoured.The selection differential (40b) simplifies to


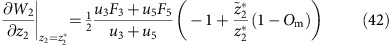


Without overlapping generations of males 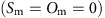 the equilibrium sex ratio is again unbiased: 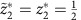. If male generations do overlap 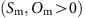 daughters are overproduced in equilibrium: 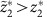, that is, a female-biased summer sex ratio.

### Sex ratio selection in diploids with female hibernation

The selection differentials (40a and b, 152) simplify to


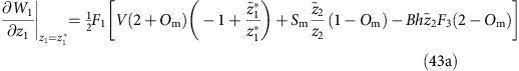



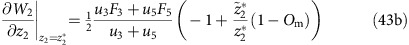


Now the overlapping-generation parameter *O*_m_ is given by equation (30). Qualitatively, the same results hold as for haplodiploids: in the absence of help (*h*=0) and with non-overlapping generations of males 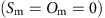, the equilibrium sex ratios are unbiased (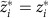). If male generations overlap 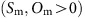, the equilibrium spring sex ratio is male-biased and the summer sex ratio is female-biased. However, again, a sufficiently large benefit from help favours female-biased sex ratios in the spring.

### Sex ratio selection in haplodiploids with larval diapause

The selection differentials (40a and b, 152) now simplify to


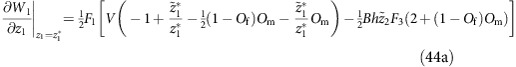



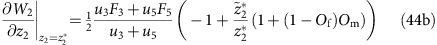


The overlapping generation parameters *O*_f_ and *O*_m_ are given by equations (34a) and (34b), respectively. In the absence of help (*h*=0), and with non-overlapping generations of males 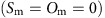, clearly the equilibrium sex ratios are again unbiased: 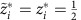. Without help but with overlapping male generations 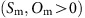, the equilibrium spring sex ratio is female-biased, that is 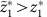, while the equilibrium summer sex ratio is male-biased, 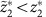, in contrast to the results for the FH life cycle. With help 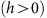, a female-biased spring sex ratio is favoured regardless of generation overlap.

### Sex ratio selection in diploids with larval diapause

The selection differentials (40a and b, 152) now simplify to


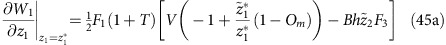



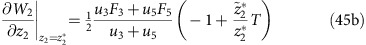


The overlapping generation parameter *O*_m_ is given by equation (34b) and T by equation (38). Qualitatively, the same results hold as for haplodiploids with the LD life cycle: in the absence of help (*h*=0), and with non-overlapping generations of males 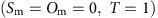, the equilibrium sex ratios are 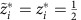. In the absence of help but with overlapping male generations 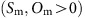, the equilibrium spring sex ratio is female-biased (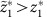), while the equilibrium summer sex ratio is male-biased (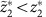). With help 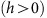, a female-biased spring sex ratio is always favoured.

### Selection on helping behaviour

The inclusive fitness of a focal daughter with helping tendency *h* in a resident population with helping tendency 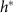 is given by


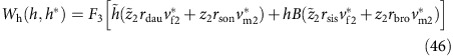


The first term between brackets is the inclusive fitness through daughters and sons obtained by not helping (with probability 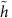), while the second term is the inclusive fitness through additional sisters and brothers obtained by helping (with probability *h*). The appropriate coefficients of relatedness and RVs depend on the specific scenario regarding genetics and life history and will be derived below.The corresponding selection differential is then:


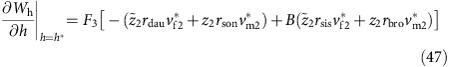


### Selection on helping behaviour in haplodiploids with female hibernation

Plugging in class frequencies, RVs and the appropriate relatedness coefficients (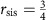, 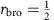), the selection differential (47) simplifies to


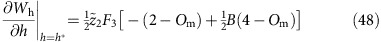


Therefore helping will be selected for whenever


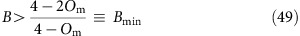


Since


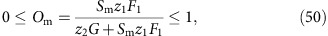


it follows that


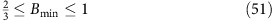


A greater generation overlap in males favours a lower benefit threshold for helping behaviour to evolve, which in turn is favoured by a male-biased spring sex ratio and female-biased summer sex ratio—precisely the sex ratios favoured by selection in the FH life cycle.

### Selection on helping behaviour in diploids with female hibernation

Using the RVs (27) and the relatedness coefficients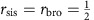, the selection differential (45) now reduces to


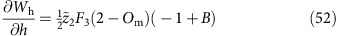


Obviously helping will be selected for whenever


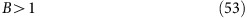


Unlike in haplodiploids, for diploids with the FH life cycle generation overlap in males does not favour a lower helping threshold and helping is always more difficult to evolve than in haplodiploids.

### Selection on helping behaviour in haplodiploids with larval diapause

Using the RVs (33c and d) and the relatedness coefficients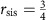 and 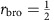, the selection differential (47) now becomes


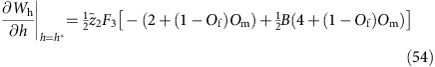


where the generation-overlap parameters *O*_f_ and *O*_m_ are given by equation (34a and b). Now helping will be selected for whenever


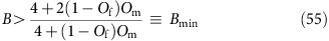


Whenever there is some generation overlap, 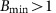 and in general


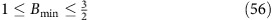


In general, therefore, helping in haplodiploids with the LD life cycle is harder to evolve than in haplodiploids with the FH life cycle.

### Selection on helping behaviour in diploids with larval diapause

Using the RVs (37c,d) and the relatedness coefficients 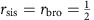, the selection differential (47) now becomes


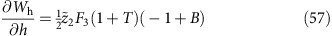


Just like in diploids with the FH life cycle, helping is selected for whenever


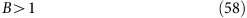


Therefore, in contrast to the FH life cycle, for the LD life cycle haplodiploidy makes helping more difficult to evolve.

### Coevolution of sex ratios and helping behaviour

Coevolution of sex ratios and helping behaviour was modelled using a standard adaptive dynamics approach^26^,^27^. For each trait *x* 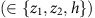, the dynamics over evolutionary time *t* is given by


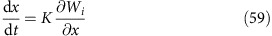


The scaling constant 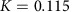 was chosen to make the adaptive dynamics results commensurate with the results from individual-based simulations (see section ‘Individual-based simulations’). The selection differentials are given above.

Numerical integration of differential equation (59) was carried out with R 3.1.0 (ref. 67), using the package deSolve^68^.

### Polyandry hampers the evolution of helping

We look at two types of polyandry: (1) serial monogamy, where surviving spring females mate for a second time with a different male; sperm from the first mating is not stored. Thus, females from the first and second brood are half-sisters, and the relatedness coefficient must be replaced accordingly 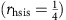. (2) Polyandry, where autumn females mate with more than one male. Their sperm is stored and used by surviving females to produce a second brood. The number of males that females mate with determines the coefficient of relatedness between females. We show calculations only for the FH life cycle.

### Serial monogamy

Since a female does not store sperm, class 6 disappears; otherwise the demography remains the same. The reproductive values are now given by


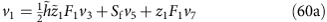



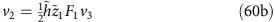



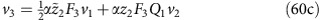



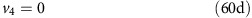



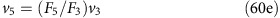



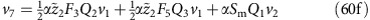


Here 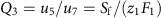. To analyse the evolution of helping we only need the RVs of summer daughters and summer sons, where we normalize the former to unity:


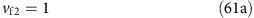



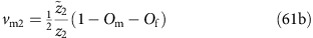


Here *O*_m_ is given by equation (24) and


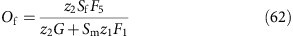


The selection differential is the same as (47) except that *r*_sis_ is replaced by *r*_hsis_, and it simplifies to


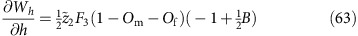


Clearly, positive selection for helping requires


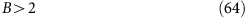


This is a much stricter condition than condition (49) under monogamy–indeed the threshold benefit is at least twice at large and at most three times as large.

### Polyandry

The relevant RVs are the same as for the monogamy scenario, (23c,d). The only difference is that the relatedness of full sisters is replaced by a relatedness coefficient (*r*_sp_) that depends on the effective number of males females mate with, and that differs between haplodiploidy and diploidy.

### Effect of polyandry on selection in haplodiploids

The coefficient of relatedness between female offspring of the same mother, for haplodiploids in a fully outbred population is given by


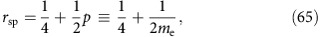


where 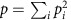 is the probability that two females with the same mother share the same father, which equals the sum of squared paternity shares *p*_*i*_ of all males that have mated with the same female. The inverse of *p* in turn defines the effective number of mates *m*_e_ per female, which is bounded above by the average number of mates per female^29^,^69^. Replacing the coefficient of relatedness (65) into the selection gradient (47) we get


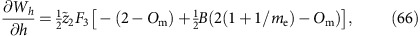


and the condition for the evolution of helping is


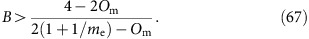


If we assume that females only mate with a single male (*m*_e_*=*1), we obtain previous the result for monogamy (49). If we assume *m*_e_≥2, then *B*_min_>1, but if 1<*m*_e_<2, then it is possible that *B*_min_<1 as long as *O*_m_>0 (Supplementary Fig. 4). In the limit of infinitely many males, such that all females with the same mother are half-sisters, we obtain the same result as in serial monogamy that helping requires helpers to be more twice as efficient (*B*_min_>2) at raising sibling as at raising offspring. Condition (67) shows that haplodiploidy can benefit the evolution of helping under the female hibernation scenario even if females are not strictly monogamous, but this effect diminishes as females are more promiscuous (Supplementary Fig. 4).

### Effect of polyandry on selection in diploids

The coefficient of relatedness under diploidy between two sisters is given by


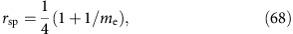


where, like before, *m*_e_ is the effective number of males females mate with. The helping selection differential then reduces to


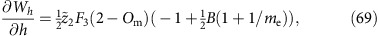


and the condition for the evolution of helping is


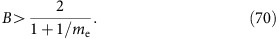


From condition (70) it is clear that if females mate with only one male (*m*_e_*=*1) we recover the previous result (53) that helpers need to be more efficient at raising their sibling than their offspring (*B*>1). If females mate with infinitely many males, the evolution of helping requires helpers to be more than twice as efficient at raising sibling as offspring (*B*>2). All in all, higher levels of female promiscuity increases the necessary benefits for the evolution of helping behaviour.

### Individual-based simulations

Evolutionary dynamics were also analysed using individual based simulations. We simulated a population that has the life cycle exemplified by Supplementary Figs 1 and 2. Each individual is characterized by three loci. The loci represent proportion of males in the spring (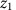) and summer (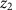), and the probability that a female born in the spring stays to become a helper (*h*). We assume that the phenotypic effect of an allele in each locus is a continuous quantity that ranges between 0 and 1. Sex ratios in the colony are in full control of the reproductive females, therefore, sex ratio loci are only expressed by reproductive females. Furthermore, all traits are expressed in codominance, thus, the phenotypic value of an individual is an average of the two allelic values. Individuals starting the season (founders) can reproduce up to two times (bivoltine life cycle), producing a spring and a summer brood. Individuals born in the spring brood can reproduce once and contribute to the summer generation. In the case of females, they can also stay and help their mother care for their siblings; this happens with a probability equal to the average allelic value of the helping locus. We assume that fecundity of a foundress in the summer is a linear increasing function of the number of helpers (see section Effect of helping). Individuals born in the summer overwinter to start the population in the following spring. Depending on the life-history, overwintering occurs only for females (female hibernation, Supplementary Fig. 1) or for both sexes (larval diapause, Supplementary Fig. 2). Generations overlap depending on the sex specific survival probability (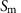 for males, and 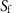 for females). Newly born females mate at random with males available at that point of the season, thus, when male generations overlap surviving males from a former brood can mate with newly born females. *N* nests (Table 2), each with one foundress, start each year. Population grows during the season by the production of both broods according to the fecundity values of females. During winter, density dependence re-establishes the population size back to the initial value.

During reproduction mutation occurs on each allele with probability *μ*. If mutation occurs, the allelic value is changed by a value drawn from a normal distribution with mean 0 and s.d. 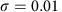 (Table 2). Allelic values for the sex ratio loci start at 0.5. In the helping loci allelic values start at 0, and mutation only occurs after 10,000 time steps, to allow for the sex ratios to reach their equilibrium values. The moment in which mutations are introduced on the helping loci does not change the qualitative results of the simulations. Moreover, parameters chosen for the mutational process do not affect the qualitative outcome of the model. We run the simulation for 25,000 time steps, each time step corresponds to one year.

### Data availability

No datasets were generated or analysed during the current study. C++ code used for the individual-based simulations can be found in Supplementary Data 1.

## Additional information

**How to cite this article:** Quiñones, A. E. & Pen, I. A unified model of Hymenopteran preadaptations that trigger the evolutionary transition to eusociality. *Nat. Commun.* **8,** 15920 doi: 10.1038/ncomms15920 (2017).

**Publisher’s note:** Springer Nature remains neutral with regard to jurisdictional claims in published maps and institutional affiliations.

## Supplementary Material

Supplementary Information

Supplementary Data 1

## Figures and Tables

**Table 1:** Parameters and variables of the inclusive fitness model.

**Symbol**	**Description**
*F*_*i*_	Fecundity class-*i* females
*S*_*f*_	Survival probability adult spring females
*S*_*m*_	Survival probability spring (LD) or summer (FH) males
*O*_*m*_	Degree of generation overlap in males
*u*_*i*_	Frequency of class-*i* individuals in demographic equilibrium
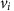	RV of class-*i* individuals
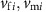	RV of resp. daughters (f) and sons (m) in spring (*i*=1) and summer (*i*=2)
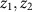	Proportion sons in spring resp. summer
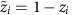	Proportion daughters
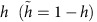	Helping tendency of spring daughters
α	Scaling factor of winter survival to ensure stable population sizes
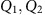	Mean no. of mates for males contributing to spring resp. summer generations
*b*	Benefit of help. Additional offspring per helper per offspring
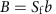	Expected benefit of help, conditional on maternal survival
*r*_*x*_	Coefficient of relatedness of *x* to female controlling evolvable trait
*p*	Probability two females with the same mother share the same father
*m*_e_	Effective number of mates per female
*W*	Inclusive fitness
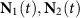	Vector of class distribution for resp. spring, summer generations in year *t*
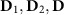	Demographic transition matrices for resp. spring, summer, overall populations
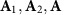	Gene flow matrices for resp. spring, summer, overall populations
*λ*	Population growth factor (dominant eigenvalue)

FH refers to the female hibernation life cycle, LD to the larval diapause life cycle, RV to reproductive value. Bold letters correspond to matrix notation, indicating an element is either a vector or a matrix.

**Table 2:** Parameter values used in the individual based simulations.

**Parameter**	**Value**	**Description**
*N*	5,000	Number of nests or founding females in the spring
μ	0.01	Mutation rate
σ	0.01	s.d. of normal distribution from which mutation values are drawn
*t*	25,000	Number of years the simulation was run
