# Supplementary material for: A unified model of Hymenopteran preadaptations that trigger the evolutionary transition to eusociality
Source: Nat Commun. 2017 Jun 23;8:15920. doi: 10.1038/ncomms15920 (PMC5490048; doi:10.1038/ncomms15920)
Supplement: Supplementary Information [file ncomms15920-s1.pdf]

File name: Supplementary Information
Description: Supplementary Figures

File name: Supplementary Data 1
Description: C++ code used for the Individual-Based Simulations

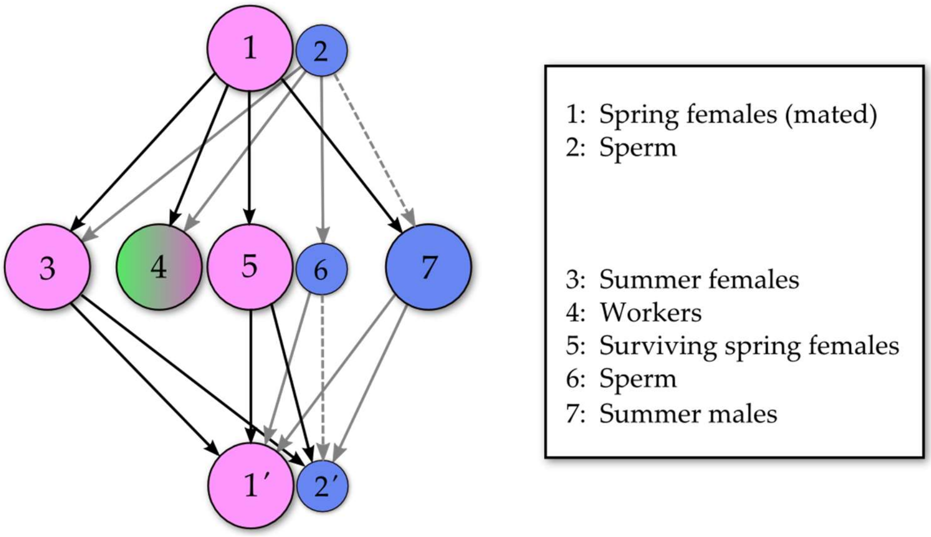

Supplementary Figure 1. Structure of the female hibernation (FH) life cycle. The top row of disks represents the spring generation of mated females (class 1) and the sperm (class 2) they store. The middle row of disks represents the summer generation consisting of non-helping daughters (class 3), helping daughters (class 4), surviving spring females (class 5) and the sperm they carry (class 6), and sons (class 7). The bottom row represents the spring generation of the next year. Solid arrows represent genetic contributions (female, black; male, grey) from one class to another through reproduction or survival for both haplodiploid and diploid species, while the dashed lines are contributions unique to diploid species.

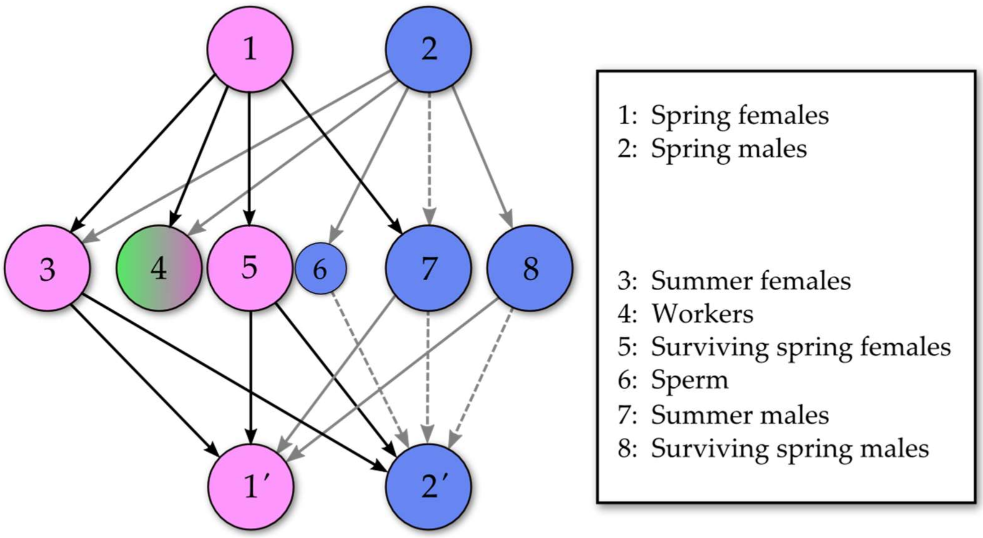

Supplementary Figure 2. Structure of the larval diapause (LD) life cycle. The top row of disks represents the spring generation of mated females (class 1) and males (class 2). The middle row of disks represents the summer generation consisting of non-helping daughters (class 3), helping daughters (class 4), surviving spring females (class 5) and the sperm they carry (class 6), sons (class 7) and surviving spring males (class 8). The bottom row represents the spring generation of the next year. Solid arrows represent genetic contributions (female, black; male, grey) from one class to another through reproduction or survival for both haplodiploid and diploid species, while the dashed lines are contributions unique to diploid species.

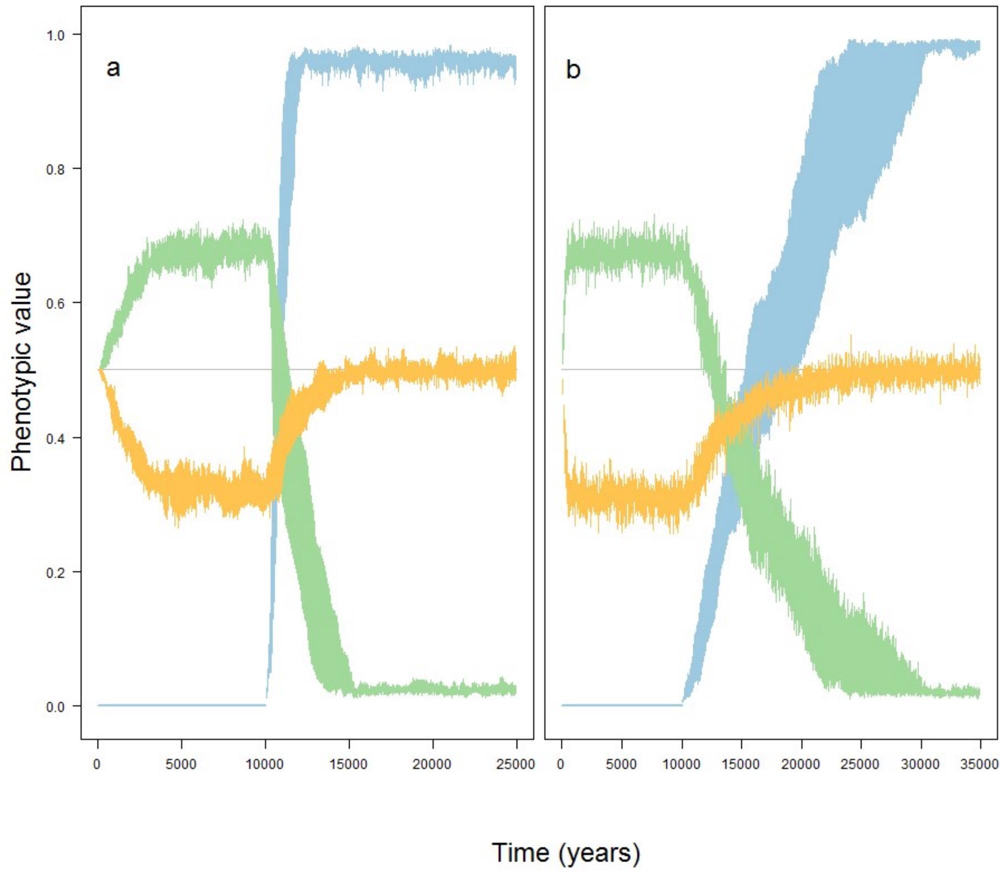

Supplementary Figure 3. Set of individual based simulations showing the robustness of the results to variations in the mutational structure of the evolving traits. Colour ribbons show ranges of 10 stochastic individual-based simulations where helping behaviour and sex ratios evolved with different mutational structures. Panel **(a)** shows simulations where mutation in the helping trait are drawn from a normal distribution with standard deviation five times higher $(\sigma_h = 0.05)$ than the mutations in the sex ratios $(\sigma_z = 0.01)$. Panel **(b)** shows simulations where the mutations in the sex ratios were drawn from a distribution with standard deviation 5 times higher $(\sigma_z = 0.05)$ than mutations for the helping trait $(\sigma_h = 0.01)$. Despite differences in how long it takes for the evolutionary transition to occur, in both cases there is a transition from solitary bivoltine to eusocial univoltine life history.

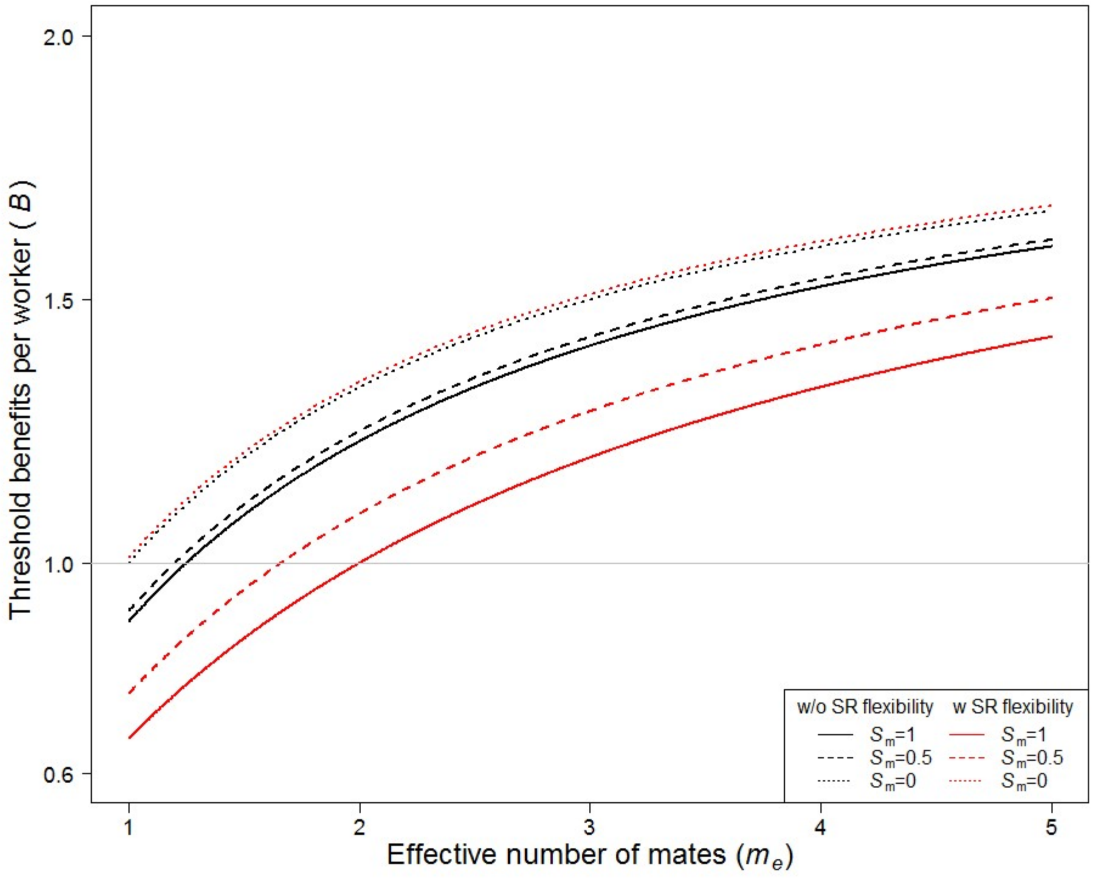

Supplementary Figure 4. Effect of polyandry on the evolution of reproductive altruism. Effect of polyandry (effective number of mates) on the threshold benefit per helper required for the evolution of reproductive altruism, for haplodiploids having female hibernation with sex ratio flexibility (red curves) and without sex ratio flexibility (black curves), at varying levels of male generation overlap (varying S_m; red [visible] and black [invisible] dotted curves overlap). For each scenario, an increasing level of polyandry always hampers the evolution of reproductive altruism. Note that some combinations of polyandry ($m_e > 1$) and sex ratio flexibility can present a more favourable scenario for the evolution of reproductive altruism than strict monogamy ($m_e = 1$) without sex ratio flexibility. As the level of polyandry increases to it theoretical maximum ($m_e \rightarrow \infty$) the threshold of helper benefits approaches 2, for all scenarios analysed.
